# Commentary on “Outcomes of Convalescent Plasma with Defined High versus Lower Neutralizing Antibody Titers against SARS-CoV-2 among Hospitalized Patients: CoronaVirus Inactivating Plasma (CoVIP) Study”

**DOI:** 10.1128/mbio.02653-22

**Published:** 2022-10-31

**Authors:** Michael J. Joyner, Nigel Paneth

**Affiliations:** a Department of Anesthesiology and Perioperative Medicine, Mayo Clinic, Rochester, Minnesota, USA; b Department of Epidemiology and Biostatistics, Michigan State University, College of Human Medicine, East Lansing, Michigan, USA; c Department of Pediatrics and Human Development, Michigan State University, College of Human Medicine, East Lansing, Michigan, USA

## Abstract

The totality of evidence favors the efficacy of convalescent plasma to treat COVID-19 when high-titer plasma is administered early in the course of disease or to immunocompromised patients. In this commentary, we frame the findings of L. A. Bartelt, A. J. Markmann, B. Nelson, J. Keys, et al. (mBio 13:e01751-22, 2022, https://doi.org/10.1128/mBio.01751-22) in the context of the normal approval process for a therapeutic product. We point out that convalescent plasma has taken all of the typical steps associated with approval for a therapeutic product. Additionally, in less than 3 years, the optimal use cases and continued utility of this product to treat COVID-19 have been defined.

## COMMENTARY

In ordinary times, therapeutic development follows a well-defined stepwise process. These steps include conceptual bioplausibility, *in vitro* demonstration of mechanism of action, and efficacy in animal models. If there are no obvious safety or toxicology concerns, human studies are the next step, including calibration of the dose of the therapeutic agent and defining the optimal use case. If things go well, large, late-phase trials are performed, which, if successful, are followed by approval of the therapeutic agent. Subsequently, real-world data become available to confirm the efficacy of the product and perhaps define additional use cases.

This process is highly regulated, is funding and market dependent, and can take a decade or more. The COVID-19 pandemic, however, forced these steps to be compressed and to be completed in parallel for vaccines, small-molecule antivirals, and monoclonal antibodies directed against severe acute respiratory syndrome coronavirus 2 (SARS-Cov-2). The pharmaceutical companies developing and studying these therapeutic agents also received massive allocations of government, philanthropic, and private funds (1).

The study of convalescent plasma (CP) as a therapeutic agent in SARS-Cov-2 has had no corporate agency behind it and has received limited funding. But two plus years into the pandemic, all of the boxes in the normal therapeutic development scheme have been checked, though not in the usual temporal sequence. Antibodies against SARS-Cov-2 harvested from convalescent donors are a conceptually bioplausible therapeutic entity as they neutralize virus *in vitro* (2). CP can be used to prevent and treat SARS-Cov-2 infection in animal models (3). Like its parent product, fresh frozen plasma, CP is safe, and with some limitations, commercial assay systems are now available to characterize CP as a therapeutic product (4, 5). Moreover, convalescent plasma and related products had an excellent track record of success and safety in the preantibiotic era (6).

Bartelt and colleagues now report the findings of what would normally have been akin to an early phase dose-finding treatment trial in humans (7). They show that hospitalized patients with SARS-CoV-2 infection for less than 10 days have better outcomes when treated with 2 U (400 to 500 mL) of high-titer plasma than patients treated with plasma with antibody titers that are lower but still above the FDA-mandated threshold.

The authors planned a randomized trial of high- versus ordinary-titer CP, but the lack of a sufficient supply of high-titer plasma required them to analyze the data as a quasi-experiment, comparing outcomes not by assignment to but by receipt of high-titer or usual plasma. Like an observational study, the findings required confirmation using multivariate methods, a test that these findings passed. The authors also present alternative ways to analyze the data, none of which materially alter the overall conclusions of the study.

A very useful table in the supplemental material contains the authors’ assessment of many convalescent plasma trial findings in relation to the antibody titer of the transfused plasma, finding that, as in this article, clinical improvement in hospitalized patients (including mortality benefit) was seen exclusively in randomized clinical trials (RCTs) that used adequate doses of high-titer plasma. The dependence of clinical benefit from CP on having an adequate amount of relevant antibody in the transfusion helps explain why several large RCTs of CP have been framed as negative. Positive well-done RCTs and confirmatory real-world data are additional boxes in the normal regulatory pathway that have been checked for CP (8–11).

The focus of Bartelt et al. on high-titer CP and the publication of these findings during the third year of the pandemic after multiple waves, multiple variants, and the approval of effective vaccines could not be more timely. CP from patients who have been both vaccinated and recovered from recent infection can now be made available worldwide at scale. This high-titer hybrid or “VaxPlasma” CP is polyclonal, “broad spectrum,” and—unlike monoclonal antibodies—resistant to escape by new SARS-Cov-2 variants (12).

The virulence of SARS-Cov-2 is now waning in much of the population whose immune systems have been primed by infection and/or vaccination. But high-titer CP has an important therapeutic niche in the treatment of immunosuppressed patients with SARS-Cov-2, many of whom are B-cell depleted and unable to generate endogenous antibody responses. They can be infected for months with newer variants that have escaped monoclonal antibodies, and CP is often the only treatment that clears their viremia (13–15).

Less than 3 years into the pandemic, both the optimal use case of CP and its optimal dose are now clear. With some attention to logistics, it should be possible to harvest and distribute this product at scale. That this has happened with modest levels of government and philanthropic support absent a major role by industry is remarkable. The article by Bartelt et al. reminds us that no therapeutic product can be successful unless we know the dose required to do the job.
